# Polysaccharide-Drug Conjugates: A Tool for Enhanced Cancer Therapy

**DOI:** 10.3390/polym14050950

**Published:** 2022-02-27

**Authors:** Neena Yadav, Arul Prakash Francis, Veeraraghavan Vishnu Priya, Shankargouda Patil, Shazia Mustaq, Sameer Saeed Khan, Khalid J. Alzahrani, Hamsa Jameel Banjer, Surapaneni Krishna Mohan, Ullas Mony, Rukkumani Rajagopalan

**Affiliations:** 1 Department of Biochemistry and Molecular Biology, School of Life Sciences, Pondicherry University, Puducherry 605014, India; neenayadav100@gmail.com (N.Y.); fdapharma@gmail.com (A.P.F.); 2 Centre of Molecular Medicine and Diagnostics (COMManD), Saveetha Institute of Medical & Technical Sciences, Saveetha Dental College and Hospitals, Saveetha University, Chennai 600077, India; vishnupriya@saveetha.com (V.V.P.); ullasmony@gmail.com (U.M.); 3 Department of Maxillofacial Surgery and Diagnostic Sciences, Division of Oral Pathology, College of Dentistry, Jazan University, Jazan 45142, Saudi Arabia; dr.ravipatil@gmail.com (S.P.); samarkhan8@gmail.com (S.S.K.); 4 Dental Health Department, College of Applied Medical Sciences, King Saud University, Riyadh 11451, Saudi Arabia; smushtaqdr@gmail.com; 5 Department of Clinical Laboratories Sciences, College of Applied Medical Sciences, Taif University, Taif 21974, Saudi Arabia; ak.jamaan@tu.edu.sa (K.J.A.); h.banjer@tu.edu.sa (H.J.B.); 6 Departments of Biochemistry, Molecular Virology, Research, Clinical Skills & Research Institute & Simulation, Panimalar Medical College Hospital, Varadharajapuram, Poonamallee, Chennai 600123, India; krishnamohan.surapaneni@gmail.com

**Keywords:** chemotherapy, cancer, polysaccharides, toxicity, drug delivery

## Abstract

Cancer is one of the most widespread deadly diseases, following cardiovascular disease, worldwide. Chemotherapy is widely used in combination with surgery, hormone and radiation therapy to treat various cancers. However, chemotherapeutic drugs can cause severe side effects due to non-specific targeting, poor bioavailability, low therapeutic indices, and high dose requirements. Several drug carriers successfully overcome these issues and deliver drugs to the desired sites, reducing the side effects. Among various drug delivery systems, polysaccharide-based carriers that target only the cancer cells have been developed to overcome the toxicity of chemotherapeutics. Polysaccharides are non-toxic, biodegradable, hydrophilic biopolymers that can be easily modified chemically to improve the bioavailability and stability for delivering therapeutics into cancer tissues. Different polysaccharides, such as chitosan, alginates, cyclodextrin, pullulan, hyaluronic acid, dextran, guar gum, pectin, and cellulose, have been used in anti-cancer drug delivery systems. This review highlights the recent progress made in polysaccharides-based drug carriers in anti-cancer therapy.

## 1. Introduction

Cancer is the most prevalent deadly disease, next to cardiovascular disease, throughout the world [1]. About 19.3 million new cancer cases and nearly 10.0 million cancer deaths were reported globally in 2020. The global survey on cancer in 2020 reported a higher incidence rate (19%) in men than women. Breast and lung cancer, the most common cancers reported, contributing to 23.1% of the new cases diagnosed in 2020. Colorectal cancer was the third most common cancer, with 1.93 million new cases in 2020 [2]. Approximately 70% of cancer deaths were reported from low- and middle-income countries. The significant causes for cancer include environmental factors, viral or genetic constituents, excessive alcohol intake, tobacco use, excessive body weight, lack of physical exercise, and low intake of vegetables and fruits. In low-income nations, nearly 25% of cancer cases are caused by hepatitis and the human papillomavirus. Genetic lesions in the genes encoding cell cycle proteins and somatic mutations in upstream cell signaling pathways may lead to cancer. Moreover, cancer cell metastasis, heterogeneity, recurrence, and their resistance to chemotherapy and radiotherapy reduce the efficacy of traditional treatment against many malignant tumors [3]. Extensive research has been carried out to develop more effective anti-cancer drugs [4]. Since the existing medications are not effective in treating cancer, the cure from cancer is considered the holy grail. Various treatments are available to treat cancer, such as surgery, radiotherapy, chemotherapy, immunotherapy and hormone therapy [5,6].

Chemotherapy is widely used, but is associated with severe side effects, including nerve problems, hair loss, weight changes, sexual dysfunction, and anemia. Moreover, chemotherapy is required in high doses for the treatment, as it is not specific for the target site. Chemotherapy mainly targets the DNA synthesis and mitosis process to kill cancerous cells [7]. Hence, conventional chemotherapy kills normal healthy cells along with cancer cells, causing severe unwanted side effects. Therefore, it is necessary to develop new chemotherapeutics that target cancerous cells without affecting normal cells. Researchers have focused on polymeric drug delivery carriers since the 1980s to improve the therapeutic index and bioavailability of conventional drugs [8]. In the last two decades, the polymeric drug delivery system has emerged as a key player in cancer treatment [9]. Polymeric carriers can be tuned through chemical modification to target the diseased site and deliver the drug in a controlled manner. Biodegradable and bioabsorbable polymers provide a safe platform for drug delivery that reduces the side effects [10]. Several biocompatible polymers, including carbohydrate-based polymers, and polysaccharides are used to improve the therapeutic efficacy of the cancer drugs and reduce the side effects. Nowadays, various immunological therapies and targeted drug delivery were included in cancer treatment based on carbohydrate polymers. Drug delivery systems based on specific targeting mechanisms will increase the efficacy of therapeutics [11]. Polysaccharide polymers are commonly used as drug carriers because of their acceptance in the body due to their biochemical and structural similarity with human extracellular matrix components. Herein, we review the current research on the polysaccharide-based carriers used in drug delivery systems [12].

## 2. Properties of Polysaccharides Carriers

Polysaccharides can be defined based on their chemical structure containing monosaccharides units linked together by glycosidic bonds [13]. They can be classified based on the monosaccharide components, chain length, and branching to the chains. The glycosidic linkage between the anomeric carbon atom of both donor and acceptor monosaccharide units distinguish them from proteins and peptides. Polysaccharides naturally possess storage properties that provide physical structure and stability, e.g., cellulose. Further, polysaccharides are differentiated into positively charged (chitosan) and negatively charged (alginate, heparin, hyaluronic acid, and pectin) based on the presence of the functional groups [14]. Chemical modifications including sulfation, phosphorylation, and carboxymethylation alter the biological properties of polysaccharides [15], making them suitable for drug carriers. They have also preferred drug carriers because of their stability, non-toxicity, and biodegradable properties. Moreover, chemical modifications subsequently improve the therapeutic efficacy of the drug through conjugation [16]. Drugs poorly soluble in aqueous solutions result in poor absorption, undergo interaction with food and enzymatic degradation, and may end up with low bioavailability [17]. Polysaccharide drug carriers improve the bioavailability of such small molecules, proteins, and peptides by enhancing their ability to permeate into tissues owing to their subcellular and submicron size. The improved cellular uptake in tissues limits the first-pass metabolism, P-glycoprotein mediated efflux and facilitates intestinal lymphatic transport [18]. The properties of polysaccharides, including their mucoadhesive properties, enhancement in absorption, flexibility to chemical modification, biocompatibility, and reduced toxicity, boost them as effective drug delivery systems. Target-specific delivery of the drug to the diseased site reduces the drug concentration required for treatment and hence enhances the drug efficacy; this, in turn, reduces adverse drug reactions by limiting the drug distribution and thereby bypassing the organs not involved in the diseased state [19].

## 3. Biopolymer Based Materials

Biopolymers are polymeric biomolecules synthesized by living organisms. Biopolymers take part in many vital functions, such as transmittance of genetic information, cellular construction, cell signalling, and drug delivery. Biopolymers are fabricated into hydrogel, aerogel and composites, which can be used for various applications, including controlled drug release, tissue engineering and wound dressing [20]. Hydrogels are 3D networks fabricated through the cross-linking of natural or synthetic polymeric materials and capable of absorbing a substantial volume of water [21]. Hydrogels are used in drug delivery systems because of their admirable properties, such as biocompatibility, biodegradability, and non-toxicity [20,22]. Nanoparticle addition into the polymeric hydrogels provides unique properties such as enhanced mechanical, thermal and magnetic properties, selectivity, and high swelling rate. Polymer-based hydrogels are widely used in biomedical applications such as tissue engineering, cell bioreactors, and drug delivery [23]. Aerogels, consisting of an ultra-lightweight structure with a porous network, can be obtained from hydrogels by removing the pore liquid using appropriate dehydration methods without any significant modification in the network structure. Biopolymers overcome the drawbacks of traditional materials used for aerogel preparation that limit their use in biomedical applications [24,25]. A study revealed the gastro-retentive properties of bendamustine hydrochloride loaded cellulose nanofiber aerogels prepared using freeze-drying. The nanofiber aerogels showed a higher area-under-the-curve (AUC) in the pharmacokinetics study in comparison with the free drug [26]. The large surface area of aerogels with an interconnected pore network facilitates the contact with the aqueous milieu and hence enhances the drug solubility [27]. Polysaccharide composites made of chitosan, alginate, cellulose, and starch are commonly used in the drug delivery system. A chitosan–polyethylene glycol (PEG) hydrogel fabricated using a silane crosslinker was pH-responsive and helpful in controlled drug delivery [28]. A pectin-based composite prepared using calcium, ethylcellulose and hydroxy propyl methyl cellulose revealed an efficient colon-specific drug delivery [29]. Carbon nanocomposites prepared using carbon nanotubes with biopolymers such as starch and chitosan have been used for drug delivery. A study reported on hollow starch nanoparticles showed an efficient delivery of DOX into hepatocellular liver cells [30]. A redox-sensitive hydroxyethyl starch doxorubicin (HES-SS-DOX) conjugate developed for anti-cancer drug delivery showed a prolonged plasma half-life time and hence exhibited better anti-tumor efficacy and reduced toxicity in comparison to free DOX [31]. Biopolymer conjugates provide a sustained drug release and help to develop time-increased release systems [32].

## 4. Polysaccharides Based Drug Carriers

Polysaccharides are carbohydrates that comprise more than ten monosaccharide units which are interlinked through a glycosidic bond. Among the natural polymers, polysaccharides have unique properties such as biocompatibility, stability, safety, adhesive properties, affinity towards the specific receptors, and non-toxicity, which make them a vital candidate in the drug delivery system [9]. Polysaccharides show a substantial structural and chemical diversity and possess a variety of functional groups that support various chemical modifications, enhancing their stability, solubility, encapsulation, and target specificity. On the other hand, the presence of a high number of the hydroxyl group in the backbone of polysaccharides encourages the inclusion of specific ligands resulting in functionalized colloidal systems. The anti-cancer potential of polysaccharides from the toxins of *B. prodigiosus* (*Serratia marcescens*) was first reviewed by Nauts et al. in 1946, and they suggested that the bacterial toxins could induce remission in cancer patients [33,34]. Polysaccharide K (PSK) and polysaccharide peptide (PSP) are protein-bound polysaccharides isolated from *Coriolus versicolor* that have been used in adjunctive immunotherapy for various types of cancer, including lung, breast, colorectal and gastrointestinal cancers [35]. Various polysaccharides used in drug delivery systems are listed in Table 1, including chitosan, alginates, cyclodextrin, pullulan, hyaluronic acid, dextran, guar gum, pectin, and cellulose.

### 4.1. Chitosan

Chitosan is a naturally occurring positively charged polysaccharide intensively used in biomedical research. Chitosan is the principal derivative of chitin, found in the cell walls of fungi, mollusk shells, and crustaceans’ exoskeletons. Chitosan is derived by deacetylation of chitin under certain conditions, and the degree of deacetylation varies from 60 to 100%. The molecular weight of commercially derived chitosan is between 3800 and 20,000 Daltons [38,39]. Chitosan contains (1 → 4)-2-acetamido-2-deoxy-β-D-glucan (*N*-acetyl D-glucosamine) and (1 → 4)-2-amino-2-deoxy-β-D-glucan (D-glucosamine) units [40]. Chitosan is a poorly water-soluble natural polysaccharide, and is soluble in a low pH solution. Modified forms of chitin, such as carboxymethyl chitin, fluorinated chitin, and sulfated glycol chitin, have been developed to improve its water solubility [40,41]. Several chemical modifications have been made to obtain many derivatives of chitosan for the controlled drug delivery system [42,43]. Chitosan shows antibacterial, antimicrobial, and anti-coagulation properties, and it also speeds up wound healing [44]. Chitosan with low molecular weight suppresses the growth of tumors and displays anti-tumor activity with a lesser toxic effect on normal growing cells [45,46]. Hence, low-molecular weight chitosan (LMWC), when used as a drug carrier, can induce synergistic effects. The cytotoxic activities of chitosan derivatives were reported in various cancer cell lines including MCF-7, HeLa and HEK293 tumor cell lines [47]. Chitosan has several features, such as cell permeability and mucoadhesive properties, which improve ocular, transdermal, and nasal drug delivery efficiency. Chitosan with a positive charge was co-assembled with negatively charged compounds to provide various types of drug carrier systems [48]. Hence, chitosan plays a major role as a drug delivery agent for low-molecular weight drugs and biomacromolecules.

Chitin and chitosan derivatives are promising candidates as polymeric carriers of anti-cancer agents. The solubility and bioavailability of chitosan are improved by derivatizing chitosan through chemical modification [43]. Previous studies have reported that the derivatization of chitosan with an acetamido residue and amino group increases the solubility of encapsulated drug molecules [12]. Some cancer cells are resistant to some anti-cancer drugs such as docetaxel (DTX), methotrexate (MTX), cisplatin, and 5-fluorouracil [49]. Currently, conventional medicines cause toxic effects on different body parts, such as the gonads, bone marrow, and gastrointestinal lining [50]. LMWC, due to its higher positively charged amino group, is highly attracted towards the cancer cell membrane with a greater negative charge than in normal cells. In addition, chitosan was reported to attack cancer cells through electrostatic interaction with the tumor cell membrane. Moreover, chitosan-drug nanoparticles can be used as alternatives to conventional drugs because of their selectivity towards the cancer cells and their biocompatibility [45,47].

#### 4.1.1. Chitosan with Doxorubicin (DOX)

DOX is an anti-cancer drug used to treat various types of cancer such as bladder cancer, breast cancer, and lymphoma. Still, DOX causes cardiotoxicity and some side effects in the human body [51]. To reduce the toxicity, DOX has been linked with chitosan by a cross-linking method that improves the anti-cancer efficacy of DOX at a lower dose [52]. DOX has been linked with chitosan by the cross-linking technique to enhance anti-cancer efficacy. The drug loading efficiency was increased by adding tripolyphosphate with a chitosan–DOX complex. The diameter of the chitosan–DOX complex was in the range of 130–160 µm. The chitosan–DOX complex showed improved anti-cancer activity in VX2 cells compared to free DOX [53]. DOX-loaded magnetic nanoparticles coated with chitosan readily entered the MCF-7 cells, accumulated around the nucleus, and delivered doxorubicin successfully. DOX-loaded nanoparticles were used as a pH-dependent drug delivery system. As they release the doxorubicin at pH 4.2, the drug can be released into the tumor environment [54]. DOX-loaded cholesterol-modified glycol chitosan micelles conjugated with folic acid significantly induced cytotoxicity against FR-positive HeLa cells [55].

#### 4.1.2. Chitosan with Paclitaxel (PTX)

**Figure 1:**
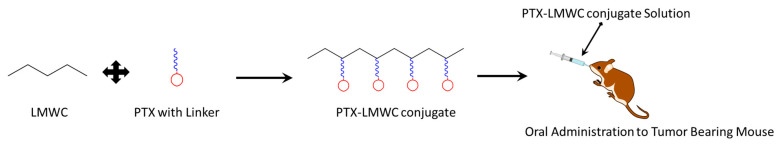
Schematic representation of the PTX–LMWC conjugate and its oral administration to tumor bearing mouse.

PTX, obtained from the Taxus brevifolia, is an effective anti-cancer drug for breast, lung, ovarian, and stomach cancer. However, the hydrophobic nature and high hemolytic toxicity of PTX are significant issues during drug delivery. Conjugation or loading of PTX with the biocompatible and biodegradable chitosan can increase the aqueous solubility of PTX and reduce the problems associated with its hydrophobic nature [56]. The PTX–chitosan complex is more efficient in cancer treatment than free PTX. The anti-cancer effect of the PTX–chitosan complex has been tested in triple-negative breast cancer cell lines (MDA-MB-231) in vitro [57]. PTX conjugated to LMWC through cleavable succinic anhydride linker enhanced its water solubility and exhibited equipotent cytotoxicity in cancer cell lines (NCIH358, SK-OV-3, MDA MB231) in comparison with free PTX. In addition, an orally administered aqueous solution of the PTX–LMWC conjugates (Figure 1) inhibited tumor growth significantly in mice bearing xenograft or allograft tumors [58]. Trimethyl chitosan PTX conjugates enhanced the mucoadhesion and intestinal transport of PTX. Additionally, the folic acid functionalization boosted the anti-cancer efficacy of the conjugate, resulting from elevated cellular uptake and intratumor accumulation [59].

#### 4.1.3. Chitosan with DTX

DTX is a chemotherapeutic drug used to treat different types of cancer, such as stomach, breast, non-small cell lung, and prostate cancer. Chitosan was modified into glycol chitosan, and DTX was loaded on the modified chitosan by means of the dialysis method. The anti-cancer action of this conjugate was tested in A549 lung cancer cell-bearing mice. The DTX-loaded glycol chitosan nanoparticles showed lesser toxicity than free DTX. The tumor-suppressing ability was also high for DTX-loaded nanoparticles than the free DTX in the case of lung cancer-bearing mice [60]. Various studies have shown that the drug-loaded glycol chitosan nano-conjugate is a promising nanoformulation in cancer treatment. DTX-loaded chitosan nanoparticles in a water-in-oil nanoemulsion system showed an enhanced cytotoxic potential in a human breast cancer cell line [61]. DTX encapsulated chitosan nanoparticles revealed higher Bax (a pro-apoptotic factor) expression over the BCL-2 (an anti-apoptotic factor) compared to the cells treated with free DTX [62].

#### 4.1.4. Chitosan with MTX

MTX was used for the first time in 1947 to treat cancer. It is used to treat breast cancer, leukemia, and lymphomas, but it causes non-specific side effects. MTX has been conjugated with natural polymers such as chitosan to reduce toxicity. Glutaraldehyde acts as a cross-linking agent to link MTX with chitosan. The ionic gelation process between the MTX, conjugated chitosan, and sodium triphosphate forms the chitosan–MTX–TPP complex. The anti-cancer efficiency of the chitosan–MTX conjugate, investigated in MCF-7, revealed that the conjugated form of MTX was more effective and less toxic than free MTX [63]. Erlotinib-loaded MTX–chitosan magnetic nanoparticles showed a thermo- and pH-dependent drug release. Selective uptake of the MTX–chitosan magnetic nanoparticles via folate receptors promoted them as a smart carrier for targeted treatment in FR-positive solid tumors [64].

#### 4.1.5. Chitosan with Curcumin

**Figure 2:**
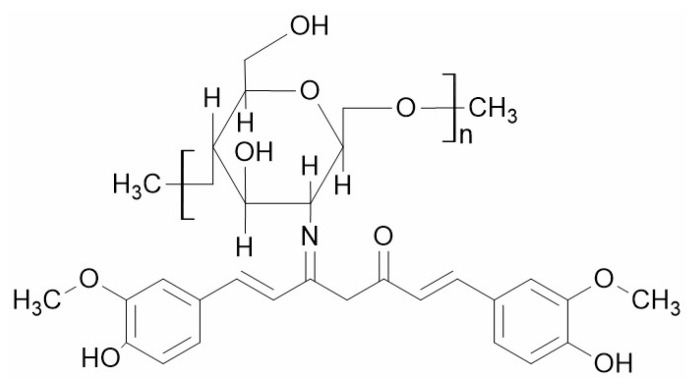
Structure of chitosan-curcumin conjugate.

Curcumin is an effective anti-cancer agent, but curcumin’s major problem is its poor pharmacokinetics profile. Chitosan-curcumin conjugate (Figure 2) obtained through the chemical conjugation of curcumin to chitosan significantly through imine formation improves the solubility and stability of curcumin. The conjugation takes places through imine bonding between the amino group of chitosan and carbonyl group present in chitosan under microwave irradiation [65]. However, a recent study showed that the introduction of 1-ethyl-3 (3-dimethylaminopropyl) carbodiimide spacer reduced the steric hindrance in curcumin conjugation, and the degree of substitution is increased by using acetate as catalyst [66]. The anti-cancer efficacy of curcumin could be increased by encapsulating the curcumin in the chitosan nanoparticles. Encapsulation of curcumin in chitosan nanoparticles increases the bioavailability of curcumin, and it works better than free curcumin. The anti-cancer efficacy of the curcumin-loaded chitosan complex has been tested in lung cancer in vitro [67]. Some in vitro studies have shown that curcumin-loaded chitosan nanoparticles were more efficient in suppressing tumor growth than free curcumin in breast cancer, hepatocellular carcinoma and colorectal cancer [68,69,70].

#### 4.1.6. Chitosan with Oxaliplatin

Oxaliplatin is a platinum-containing chemotherapeutic drug used to treat rectal and colon cancer. It suppresses or slows down the growth of the tumor. Oxaliplatin was loaded on chitosan to make a pH-sensitive nanocarrier for target specific delivery in tumor cells. Oxaliplatin-loaded chitosan nanoparticles increase the therapeutic efficiency of oxaliplatin in cancer treatment. The pH sensitive chitosan-oxaliplatin releases oxaliplatin much more rapidly at pH 4.5 than pH 7.4, which is essential for tumor-targeted drug delivery [71]. The anti-cancer activity of the complex was tested in MCF-7 cell lines. The chitosan–oxaliplatin complex affects the apoptotic pathway, and it increases the expression of cytochrome C, Bax, Bik, and caspases 3 and 9 in breast cancer cells. So, the oxaliplatin–chitosan complex could be a clever approach in cancer treatment. Overall, chitosan-based carriers could be a promising strategy for delivery of anti-tumor drugs.

### 4.2. Alginate

Alginates are linear unbranched anionic polysaccharides found in the cell walls of brown algae, including *Laminaria* and *Ascophyllum*, consisting of (1 → 4′)-linked β-d-mannuronic acid and α-l-guluronic acid residues. Alginate is mainly used in the food industry, pharmaceutical, and industrial applications. Due to its properties including bioavailability, biocompatibility, low toxicity, relatively lesser cost, and moderate gelation by addition of divalent cations, alginate could be a good drug delivery agent [72]. Alginate has broad applications in cell transplantation, wound healing, and acts as a carrier for delivering proteins and small chemical drugs.

The synthesis of alginate hydrogels is straightforward and is performed by the cross-linking method. Alginate hydrogels are easily attached to mucosal surfaces due to their bio-adhesive nature and can be orally given or injected into the body directly. Such properties allow the extensive use of alginate in the pharmaceutical industry [72]. Nanoparticles of alginate can be stabilized by adding cationic polyelectrolytes [73]. The properties of alginate, including as a thickening, stabilizing, and gel-forming agent, promote it as a useful drug carrier.

Controlled release drug delivery systems are considered to give a steady and kinetically expectable drug release. Alginate, as with hydrocolloids, has played a significant role in the design of controlled-release products. Many drugs have been loaded into alginate matrices in the form of microsphere, tablets, beads, and tablets for control release therapies. Alginate nanoparticles have been used to deliver various drugs such as metformin [74], DOX [75], ethionamide [76] and several other drugs. Various administration routes of alginate nanoparticles are pulmonary [77], oral [75], nasal [78,79], intravenous [80], vaginal [81] and ocular [82].

#### 4.2.1. Alginate with DOX

**Figure 3:**
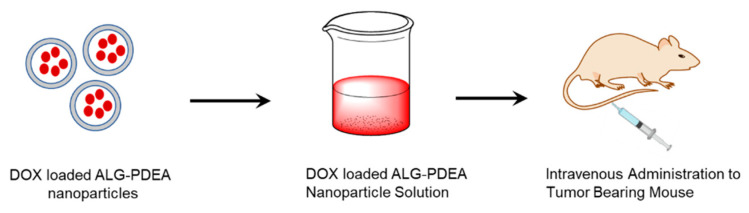
Schematic representation of DOX-loaded nanoparticles and their administration to tumor-bearing mouse.

Alginate is used in anti-cancer drug delivery systems in several forms, such as nanogels, hydrogels, and nanoparticles, due to its biocompatible and biodegradable nature [83]. Alginate nanoparticles were synthesized by the gelation of alginate with divalent cations such as calcium and loaded with DOX [84]. DOX shows a greater affinity towards alginate polymers. Alginate nanoparticles are used as a drug carrier due to their high drug loading capacity for DOX. This could be more than 50 mg per 100 mg of alginate. Experimental studies have reported that DOX-loaded nanoparticles are used to treat liver metastasis in mice. DOX-loaded alginic acid/poly(2-(diethylamino)-ethyl methacrylate) nanoparticles were tested in H22 tumor-bearing mice. Alginate nanoparticles showed more permeability and retention effect, targeting the tumor cells passively, which was confirmed by the near-infrared (NIR) fluorescence imaging technique. An intravenously administered DOX-loaded alginic acid/poly(2-(diethylamino) ethyl methacrylate) (ALG-PDEA) nanoparticle solution was more effective in H22 tumor-bearing mice than free DOX (Figure 3) [85].

#### 4.2.2. Alginate with Curcumin

**Figure 4:**
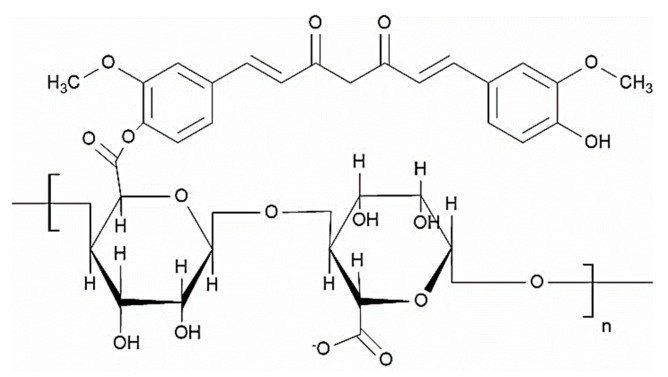
Structure of the alginate–curcumin conjugate.

Curcumin is a naturally occurring polyphenolic compound used in the treatment of different types of cancers, such as cervical, bladder, prostate and breast cancer [86]. However, the major problem is its low water solubility and poor bioavailability. This can be overcome by the entrapment of curcumin in hydrophilic calcium alginate nanoparticles and delivering it to the target site. The percentage entrapment efficiency of alginate for curcumin was reported as 49.3 ± 4.3. The curcumin-loaded calcium alginate nanoparticles were synthesized by emulsification and cross-linking method. The particle size was found to be 12.53 ± 1.06 nm. An alginate nanoformulation was checked in prostate cancer, and it showed cytotoxic effects on DU145 prostate cancer cells in vitro [87]. Moreover, the aqueous solubility of curcumin was significantly enhanced through conjugation with alginate (Figure 4). The conjugates were developed via the esterification reaction between the hydroxyl group of curcumin and C-6 carboxylate group of sodium alginate. The cytotoxic studies using L-929 mouse fibroblast cells revealed that the cytotoxic potential of curcumin was retained even after conjugation [88].

#### 4.2.3. Alginate with Exemestane (EXE)

EXE is a hydrophobic steroid aromatase inhibitor with high lipophilicity. It has shown excellent solubility in organic solvents. EXE is an oral chemotherapeutic drug mainly used to treat breast cancer by inhibiting the synthesis of estrogen [89]. It is loaded in alginate nanoparticles by simple controlled gelation methods. The loading and unloading were confirmed by XRD, SEM, and FTIR techniques. The XRD studies were performed to check the EXE encapsulation [90].

The anti-cancer activity of the formulation has been tested in vitro using DLA cells (Dalton’s lymphoma ascites). EXE has been released from the nanoformulation at pH 7.4 in vitro, and the side effects of chemotherapy are reduced by the controlled release of EXE loaded in alginate nanoparticles. Thus, in vitro studies have shown that the EXE–alginate nanoformulation could be an excellent anti-cancer agent.

#### 4.2.4. Alginate with Tamoxifen (TMX)

TMX has been widely used to treat breast cancer [91]. It shows poor solubility in water, which restricts the oral administration of the drug. This problem can be overcome by means of the use of a nanoparticle carrier system for drug delivery. Nanoparticles with bovine serum albumin and thiolate alginate have been prepared by the coacervation method and loaded with TMX. In vitro release studies of this formulation showed 45–52% TMX release after up to 25 h. Cellular uptake of TMX-loaded nanoparticles was examined in monoculture of MCF7 cells and HeLa cells [92]. The TMX-loaded alginate nanoparticles were found to be effective in both cell lines. Folate targeted alginate–silver nanoparticles loaded with TMX provides better cytotoxic effects in breast cancer cell lines. The cytotoxicity was achieved by inducting ROS, down regulating the survival oncogenic genes (BCL-2 and survivin) and arresting G2/M phase [93].

### 4.3. Pectin

Pectin is a linear polysaccharide found in the middle lamella of plants in higher concentrations [94]. Pectin comprises D-galacturonic acid (GalA) units, which are mainly joined by an α (1 → 4) glycosidic bond (homopolymer of (1 → 4) a-D-galactopyranosyluronic acid units with varying degrees of carboxyl groups methyl esterified). Rhamnose is also present in the pectin backbone, and galactose, xylose, and arabinose are present in the side chain [94,95]. The most crucial property of pectin is its gel-forming ability. Due to this property, it has been used in the food and pharmaceutical industries. The primary source of pectin is plant residue after juice extraction—the peel of citrus fruits mainly contains around 20–30%, and 10–15% is present in apples. Pectin is also obtained from other sources such as mango waste, legumes, cabbage, sunflower heads, and sugar manufacturing waste. Pectin has been widely used as a drug carrier due to its gel-forming property in acidic media. The gel-forming ability varies according to the molecular composition, molecular weight, and source from where it obtained. Pectin is generally used as a food additive, and it is entirely safe as a food additive.

Nowadays, pectin is used as a drug carrier owing to its advantageous properties such as non-toxicity, bioavailability, and low manufacturing cost. There are different modes of delivery, including administration through the nasal and oral cavity. Because of its carboxylic acid functional group, pectin is easily conjugated to the amino group of the anti-cancer drugs [96]. The anti-cancer drug from the pectin conjugate is quickly released in the tumor cell because the lysosomal enzymes easily hydrolyze the amide bond.

#### 4.3.1. Pectin with Curcumin

**Figure 5:**
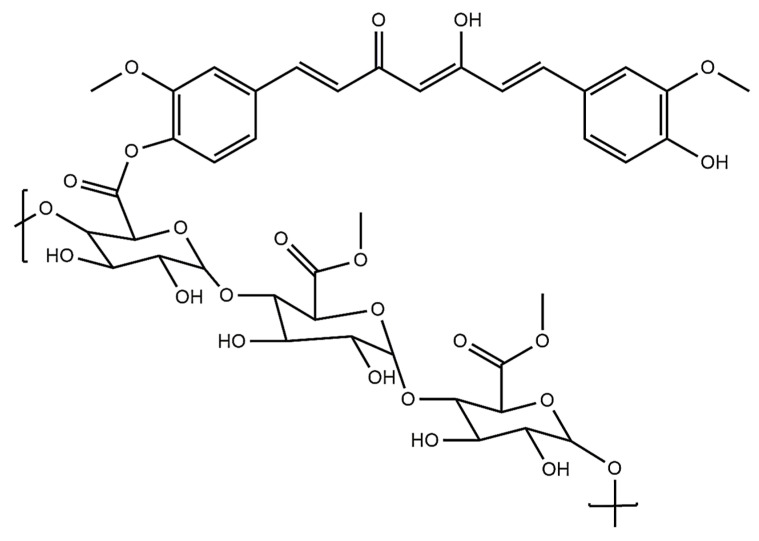
Structure of the pectin–curcumin conjugate.

Curcumin is obtained from the turmeric rhizomes and used as an anti-inflammatory, antimicrobial, antiviral, and anti-cancer agent [97]. Curcumin shows excellent hepatoprotective effects [98]. Curcumin is an ideal anti-cancer drug candidate due to its antioxidant and anti-inflammatory properties, but its poor bioavailability is the major problem during oral administration. This problem can be overcome by forming the appropriate curcumin nanoformulation with pectin. Curcumin works against human colon cancer, inhibiting cancer cell growth by modulating the NF-𝜅B signaling pathway [99]. Curcumin-loaded pectin showed a more cytotoxic effect in colon cancer (HCT116) than free curcumin. Pectin maintains curcumin’s integrity and bioactivity and delivers it to the target site in the colon cancer cell. Pectin-type B gelatin curcumin conjugates have been more effective for curcumin delivery through oral administration in anti-cancer agents [100]. Pectin and curcumin were conjugated via the esterification reaction between carboxylic groups of pectin and phenolic –OH group of curcumin (Figure 5). Cytotoxicity studies of the conjugates showed significant inhibition of KYSE-30 cell lines compared to free curcumin [101].

#### 4.3.2. Pectin with DOX

Pectin has been used as a carrier for anti-cancer drugs such as DOX. Pectin has been linked with DOX in thiolate form to obtain thiolate pectin–DOX conjugate and tested for its anti-cancer activity in human prostate cancer, human bone osteosarcoma cells and colon cancer in vitro. The thiolate pectin–DOX conjugate showed more anti-cancer activity than free DOX in the case of 143B and CT26 cells, but no significant difference was seen between the free DOX and thiolate pectin-DOX in the case of the prostate cancer cells. Thiolated pectin–DOX conjugates have been used in targeted drug delivery in CT26 cells [102]. Self-assembled DOX-conjugated hydrophilic pectin nanoparticles loaded with hydrophobic dihydroartemisinin revealed a quick release of DOX and DHA in a weakly acidic environment. The formulation significantly reduced the tumor growth in a female C57BL/6 mouse model [103].

#### 4.3.3. Pectin with Cisplatin

Cisplatin is used as an anti-cancer drug against various cancers, including lung cancer, head and neck cancer, ovarian cancer, and bladder cancer [104]. Cisplatin can be given alone or along with other cancer therapy such as radiotherapy. Pectin conjugates with cisplatin were tested in B16 cells (murine melanoma) in vitro. These in vivo studies reported the antitumor efficacy in mice. The anti-tumor efficacy was checked by measuring tumor growth. Cisplatin reduced the tumor size effectively, as studied by tumor regression studies [105]. Nano-conjugates of pectin and cisplatin were more effective when given along with radiotherapy than cisplatin. Pectin–cisplatin nano-conjugates revealed a prolonged blood retention profile in mice, which was confirmed by the presence of cisplatin in the circulation after 24 h. The J-774 cells incubated with the nano-conjugates tagged with FITC showed an uptake in 40% cells followed by 30 min of incubation [106].

### 4.4. Guar Gum

Guar gum is a naturally occurring high-molecular weight and uncharged carbohydrate mainly available in *Cyamopsis tetragonolobus*. Guar gum is made up of galactomannan, which consists of galactose and mannose. The presence of multiple hydroxyl groups makes it an excellent candidate for derivatization. Guar gum is mainly used as a stabilizing and emulsifying agent. The vital characteristic of guar gum is its swelling property, used to control drug release. Guar gum is soluble in water but insoluble in hydrocarbons, fats, alcohol, esters, and ketones [107]. It generally forms a viscous colloidal solution in both hot and cold water. Guar gum has many pharmaceutical applications, mainly in drug delivery, due to its stability, non-toxicity, and biodegradability. Guar gum and its derivative have been used as drug carriers in targeted drug delivery systems. Guar gum has been used to control the release profile of the drugs that are highly water-soluble and have difficulty in delivering at the targeted site [108].

#### 4.4.1. Guar Gum with 5-Fluorouracil (5FU)

5FU is the drug used to treat colon cancer [109]. 5FU is poorly absorbed in oral administration and hence shows low bioavailability. To improve the bioavailability, a microsphere that consists of guar gum and sodium borate was prepared by the emulsification cross-linking method. This helped to control the release of the drug for 24 h, specifically in the colon site, and it worked better against colorectal cancer. The microspheres loaded with 5FU showed more stability, and the release of 5FU followed zero-order kinetics with both degradation and erosion mechanisms. The microspheres could release the maximum amount of drug in colon cancer in a controlled manner. The significant advantages of targeting the colon for 5FU drug delivery are the pH range, which is around 5.5–7 in the colon, and the low digestive enzymatic activity, which supports more drug absorption. The effectiveness of formulation in colon-targeted oral drug delivery systems has been studied in vivo [110].

#### 4.4.2. Guar Gum with Tamoxifen Citrate (TMX)

TMX shows an antagonistic effect in breast cancer cells and is used for the treatment of ER (+) tumors [111]. Guar gum containing TMX nanoparticles was formed by the emulsion process. Guar gum has been used as a drug carrier for different anti-cancer drugs, but very little information is reported about this nanoformulation. Guar gum nanoparticles loaded with TMX was analyzed in albino mice for two days. After two days, nanoparticles were found in both mammary and ovary tissue, but the uptake and retention of nanoparticles were found to be more in mammary glands [112,113].

#### 4.4.3. Guar Gum with MTX

Guar gum microspheres were prepared and used to treat colorectal cancer [107]. The entrapment efficiency of MTX-loaded microspheres was found to be 75.7%. Guar gum microspheres delivered the maximum amount of drug at the targeted site in colon cancer. The formulation was given orally to albino rats and checked for drug release in different parts, such as the stomach, colon, and small intestine, at different time intervals. The study revealed that guar gum microspheres released MTX at the target site in the colon. Thus, guar gum microspheres have been used as an effective system for MTX delivery in the case of colorectal cancer [114].

#### 4.4.4. Guar Gum with Curcumin

Curcumin has been used as an anti-cancer drug due to its antioxidant properties, but its poor absorption in GIT is the major problem [115]. Curcumin has been formulated with guar gum of different concentrations to overcome this problem, and the formulations were investigated for their effect on colon cancer. The drug release of the formulation with 40% guar gum containing preparation was 91.1%, while the 50% guar gum containing formulation showed a drug release of 82.1%, which is lesser in comparison to the preparation containing 40% guar gum [115]. Guar gum’s effectiveness and drug release assays against colonic bacteria have been studied with rat cecal contents. Guar gum could be a promising drug carrier for curcumin delivery in colon cancer [108].

### 4.5. Dextran

Natural polymers have been used as carriers in drug delivery systems due to their non-toxicity and low production cost. Dextran is one of the natural polymers in targeted drug delivery systems [116], which contains a monomeric α-D-glucose unit and has α-(1 → 6) glycosidic linkage in the backbone. Dextran is widely used in pharmaceutical, food, and chemical industries as a carrier, stabilizer, and emulsifying agent [117,118]. Due to its properties such as biocompatibility, non-immunogenicity, non-toxicity, it has been used in drug delivery systems. It can also be easily modified and hence widely used in drug delivery systems. It increases the stability of the drug and prevents the drug from accumulating in the blood [119]. Dextran inhibits the growth of the tumor, and it reduces the toxic effects induced by the drug in the body. Dextran can be a promising drug delivery agent for anti-cancer drugs.

#### 4.5.1. Dextran with DOX

Dextran is a superior carrier in drug delivery systems, increasing the stability of DOX [120]. Mixtures of dextran–DOX and dextran–CPT showed remarkable anti-cancer activity against 4T1 cell line and 4T1 tumor-bearing mice compared to the free drugs [121]. A biocompatible poly pro-drug based on dextran–DOX prodrug (DOXDT) was formed by one-step atom transfer radical polymerization (ATRP). The drug loading capacity of DOXDT prodrug is higher than other lipid-based drug delivery systems, at up to 23.6%. The DOXDT shows improved cytotoxicity against 4T1 and HeLa cells. DOXDT was also studied for tumor-suppressive effects. The DOXDT-based delivery system suppressed the growth of tumor cells [122].

#### 4.5.2. Dextran with PTX

PTX, or Taxol (TXL), is used as an anti-cancer drug in different cancers such as breast, ovarian, and lung cancer. TXL and its derivatives were linked with aminated dextran to form dextran–TXL conjugates and were analyzed for the anti-cancer activity in HeLa-KB cells in vitro. Dextran–TXL conjugates showed two to three times greater anti-cancer effects when linked with folic acid. So, conjugation of TXL with dextran and folic acid may improve the anti-cancer efficacy of Taxol [123]. A Dex–SS–PTX conjugate showed significant cytotoxicity in BT-549 and MCF-7 cells [124].

#### 4.5.3. Dextran with Phenoxodiol (PXD)

PXD is a synthetic analog of the plant isoflavone genistein with improved anti-cancer efficacy [125]. PXD, an anti-tumor agent, was conjugated with dextran to improve its effectiveness [126]. The anti-proliferative activity of this conjugate was tested in glioblastoma, breast cancer MDA-MB-231, and neuroblastoma SKN-BE (2)C cells. The anti-cancer activity was also tested in HMEC-1 (human microvascular endothelial cells) and non-malignant human lung fibroblast MRC-5 cells. This conjugate was more stable, effective and less toxic than the free drug [127].

#### 4.5.4. Dextran with MTX

MTX is used to treat cancer and other hematological diseases. MTX is conjugated with dextran by covalent linkage to obtain a better formulation of the drug [128]. The anti-cancer activity of the conjugate was evaluated in human brain tumors (H80) and 9L gliosarcoma in rat brains. The conjugate killed the tumor cells more effectively than free MTX [129].

#### 4.5.5. Dextran with Curcumin

**Figure 6:**
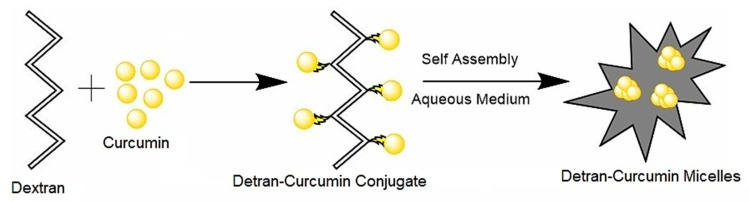
Schematic diagram depicting self-assembly of curcumin–dextran micelles.

Curcumin is actively used in cancer treatment due to its well-known biological properties. A study on carboxymethyl dextran-coated liposomal curcumin revealed improved cytotoxicity and enhanced cellular uptake in HeLa cells [130]. Dextran-curcumin micelles fabricated (Figure 6) using self-assembly of dextran curcumin conjugate displayed significant cytotoxicity in cancer cells owing to the enhanced solubility and efficient cellular internalization compared to free curcumin [131]. Curcumin has been conjugated with dextran successfully by a free radical reaction. The anti-cancer activity of curcumin–dextran conjugate has been tested in MCF-7 and adenocarcinomas gastric cell line by MTT assay. The results show that curcumin–dextran conjugates have anti-proliferative effects in MCF-7 cell lines and human gastric adenocarcinoma cells [132]. Curcumin–dextran conjugates act as a carrier for MTX and act synergistically to enhance the anti-cancer efficacy in MCF-7 cell line [133].

### 4.6. Hyaluronic Acid (HA)

HA is a mucopolysaccharide that consists of two saccharide units, glucuronic acid and *N*-acetylglucosamine [134]. HA can be easily modified due to its hydroxyl and carboxylic groups, as well as an *N*-acetyl group. Different drugs can be directly linked with HA to generate a new conjugate with more anti-cancer activity. HA is more specific to several tumor cells, particularly tumor-initiating cells. Tumor cells overexpress the CD44, LYVE-1 receptors, which are HA-binding receptors, and low expression was also seen in the surface of epithelial hematopoietic and neural cells [135]. Due to its properties such as biocompatibility, high viscoelasticity, and biodegradability, HA is used as a drug carrier in drug delivery systems in the form of hydrogel and micelles. The intracellular uptake of HA-drug conjugates was facilitated via CD44 caveolae-mediated endocytosis on tumor cells, thus enhancing the targeted drug delivery efficiency [136,137].

#### 4.6.1. HA with PTX

**Figure 7:**
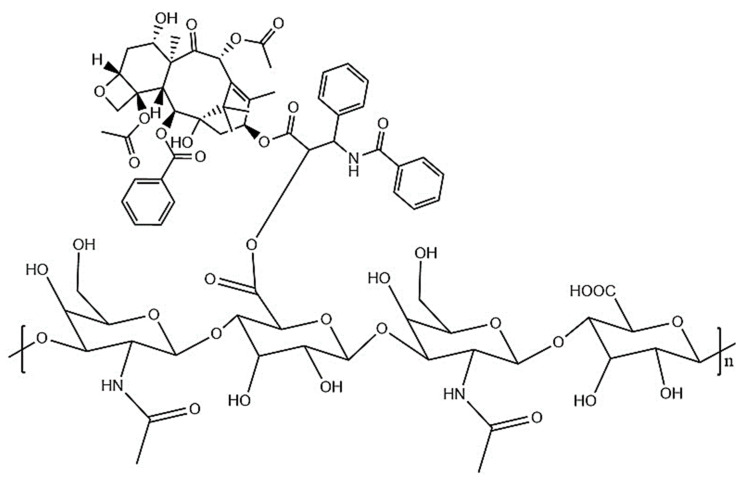
Structure of the HA–PTX conjugate.

PTX is obtained from the bark of Pacific yew, and it shows antimitotic activity. PTX is used as an anti-cancer agent, and it promotes tubulin assembly. PTX has been linked with hyaluronic acid through esterification. It inhibits the replication of cells in the late G2/M phase [138]. Hyaluronic acid was first dissolved in DMSO with polyethylene glycol (PEG) then conjugated with PTX by ester linkage without any modification [139]. The conjugate of HA-PTX forms micelles in an aqueous solution. Tumor cells over-express the hyaluronic acid receptor, and hence the HA–PTX conjugate shows more binding and cytotoxic effects in tumor cells than normal cells. The anti-tumor activity of this conjugate has been proved in MCF-7 and HCT-116 cancer cells in vitro [140]. Chitosan-coated HA–PTX nanoparticles were prepared by coating chitosan onto the surface of self-assembled HA–PTX conjugates (Figure 7). HA–PTX NPs displayed higher cellular uptake than free PTX in HepG2 cell lines [141].

#### 4.6.2. HA with DOX

DOX is used effectively to treat ovarian, breast, multiple myeloma, and pediatric solid tumors [142]. DOX has been conjugated with hyaluronic acid through amide linkage between the carboxylic acid group of HA and the amine of Dox to form an amide bond to form a nano-conjugate with improved anti-cancer activity. The anti-cancer activity of the hyaluronan–DOX nano-conjugate has been tested in MDA-MB-231 breast cancer cell lines. The nano-conjugate exhibited improved cytotoxicity in breast cancer cell lines. The conjugate showed minimum toxicity to the normal cells. The nano-conjugate also inhibited breast cancer in vivo and improved the survival rate. The nano-conjugate inhibited tumor growth at early stages in breast cancer [143].

#### 4.6.3. HA with Cisplatin

Cisplatin is generally used to treat bone and blood vessel cancer [68]. Cisplatin is an effective anti-cancer agent, but its use is limited due to its toxic effect on the nervous system. In order to reduce the harmful impact of cisplatin, it has been linked with hyaluronic acid to form a less toxic conjugate with improved tumor-targeted delivery. The conjugate has been delivered in squamous cell carcinoma of the head and neck in vivo to evaluate the anti-tumor activity. The results show the improved anti-cancer efficacy of the conjugate with lesser toxicity. The anti-cancer activity of the conjugate was also shown in dogs with soft tissue carcinoma [144].

#### 4.6.4. HA with Camptothecin (CPT)

CPT is obtained from *Camptotheca acuminate*, a Chinese tree, and it shows anti-tumor activity by inhibiting the nuclear enzymes. CPT is an alkaloid that shows poor solubility in water, so the cellular uptake is less. Several analogs of CPT have been developed to overcome the problem and improve water solubility [145,146]. 7-ethyl-10-hydroxy camptothecin is a CPT analog that has been linked with hyaluronic acid, and the resulting compound is ONCOFID-S. Its anti-cancer activity has been tested in breast cancer, esophageal, ovarian, gastric, and lung cancer [147]. Recently the anti-cancer activity of ONCOFID-S has been proved in mice with peritoneal carcinomatosis from esophageal, colorectal, and gastric adenocarcinomas [148].

### 4.7. Cyclodextrin

Cyclodextrin (CD), made of cyclic oligosaccharides, contains a hydrophilic outer layer with (α-1,4)-linked α-d-glucopyranose units and a lipophilic central cavity [149]. Cyclodextrins are biocompatible have low toxicity and low immunogenicity. The molecular weight of cyclodextrins is in the range of 1000 to 2000 Da. The example of some natural cyclodextrins is α-cyclodextrin (αCD) with six glucopyranose units, β-cyclodextrin (βCD) with seven glucopyranose units, and γ-cyclodextrin (γCD) with eight glucopyranose units. The most abundant cyclodextrin is β-cyclodextrin, which is used in the pharmaceutical industry due to its low production cost, bio-availability and perfect size cavity. Cyclodextrins form water-soluble complexes with many poorly soluble compounds. Cyclodextrins are hollow truncated, with a cavity that is slightly hydrophobic inside and hydrophilic outside. The drugs with hydrophobic nature can be easily encapsulated into the cavity of cyclodextrins to form an inclusion complex without any chemical reaction. As a result, encapsulating the drug in the cyclodextrin improves the hydrophobic drug’s stability and aqueous solubility. Cyclodextrin provides the shielding effect and reduces the side effects of the drug on the human body [150].

#### 4.7.1. Cyclodextrin with DOX

DOX, an anti-cancer drug, was entrapped in pegylated liposomes and conjugated with γ-cyclodextrin and was tested for the anti-cancer effect in BALB/c mice bearing colon-26 tumor cells. The anti-tumor activity was compared in different conjugates such as pegylated liposomes entrapping DOX, γ-CD, and the binary system of liposomes with both DOX and CD and free DOX. Various preparations have been tested in mice, and the results show that the complex-in-liposome with both DOX and CD provided high DOX levels in plasma and solid tumors compared with the other formulations. Furthermore, the results obtained from the above studies show that pegylated liposomes entrapped with DOX and γ-CD retarded tumor growth and improved the survival rate of mice, hence increasing the anti-cancer efficacy of DOX [52].

#### 4.7.2. Cyclodextrin with CPT

CPT is a hydrophobic anti-cancer drug, but its clinical application is less widespread due to some properties such as instability in physiological conditions and poor solubility in aqueous solutions. The solubility and stability have been increased by nanoparticulate systems of amphiphilic cyclodextrins, poly-epsilon-caprolactone, or poly(lactide-co-glycolide) (PLGA) [151]. The drug-loading capacity and anti-cancer efficacy were found to be greater for amphiphilic cyclodextrin nanoparticles compared to others. The anti-tumor activity of nanoparticles was tested in the breast cancer cell line MCF-7. CPT-loaded cyclodextrin nanoparticles acted as an excellent carrier system for the effective delivery of CPT [152].

#### 4.7.3. Cyclodextrin with Curcumin

Curcumin is used as an anti-cancer agent, but its poor oral bioavailability is the major problem that can be overcome by conjugating with cyclodextrin [153]. The curcumin–cyclodextrin complex has been investigated in different anti-cancer studies. In one study, curcumin was encapsulated in the β-cyclodextrin cavity using the saturated aqueous solution method. Cyclodextrin increased the delivery of curcumin, and its therapeutic value increased in vitro. It regulated various pathways such as up-regulated p53/p21 pathway, down-regulated CyclinE-CDK2 combination, increased Bax/caspase 3 expressions MAPK/NF-κB pathway and CD15. The cyclodextrin–curcumin complex has been used to improve the curcumin anti-cancer efficacy and delivery in lung cancer [154].

#### 4.7.4. Cyclodextrin with PTX

PTX is used to treat lung, breast, esophageal, bladder, and ovarian cancer. Its solubility in an aqueous solution is very low, which is a major problem in treatment. The problem can be overcome by conjugation of PTX with cyclodextrin. The anti-cancer activity of the PTX–cyclodextrin conjugate has been proved in MDA-MB-231 breast cancer cells [155]. PTX-loaded cyclodextrin peptide (R8-CMβCD) enhanced the cellular uptake of PTX by reducing the efflux of PTX in tumor cells by inhibiting P-gp efflux pump. PTX-conjugated β-cyclodextrin polyrotaxane exhibits significantly superior potency in reducing tumor growth, and enhances the lifetime of tumor-bearing mice [156,157].

### 4.8. Pullulan

Pullulan is a biopolymer made of maltotriose units joined via α (1 → 4) glycosidic bond, and successive maltotriose units were linked by α (1 → 6) glycosidic bond. Pullulan was obtained from the fermentation medium of the *Aureobasidium pullulans* [158] Pullulan is used as a drug carrier broadly due to its high aqueous solubility. A number of derivatives of pullulan were produced by some chemical modifications with different solubilities. Pullulan has not shown any toxicity to cells, and it is a non-immunogenic polymer that is useful in biomedical applications. Pullulan showed higher degradation than dextran in serum. Pullulan has been used in different ways due to its elasticity and thermal stability. The derivatization of pullulan has been performed either with DOX or DOX and folic acid. The activation of pullulan was achieved by means of periodate oxidation and was functionalized by reductive conjugation with cysteamine and PEG (NH_2_)_2_. The DOX–pullulan bioconjugates have been used in passive tumor targeting. Pullulan has been conjugated with different anti-cancer drugs for improving the anti-cancer efficiency of the drugs [159].

#### 4.8.1. Pullulan with DOX

DOX is used as an anti-cancer drug that inhibits the synthesis of DNA in cancer cells. Pullulan can be used as a drug carrier due to its properties such as biocompatibility and non-immunogenicity. Pullulan has a number of functional groups, which is a useful feature in drug delivery [160,161]. DOX shows toxic effects on normal cells that can be reduced by the encapsulation of DOX. Some studies reported that encapsulated DOX nanoparticles holding folic acid were more efficient in suppressing the tumor than free DOX. Folic-acid-conjugated pullulan-g-poly (Lactide-co-glycolide) (PLGA) copolymer has been synthesized to deliver DOX at the target site in tumor cells. Therefore, pullulan and the different pullulan derivatives such as pullulan/PLGA graft copolymer would be the perfect candidates for the fabrication of drug targeting carriers. The anti-cancer activity of the conjugate was shown in KB tumor cells in vitro [162].

#### 4.8.2. Pullulan with Mitoxantrone

Mitoxantrone is an anti-cancer drug that inhibits topoisomerase and can intercalate DNA. Due to its toxicity, the uses of mitoxantrone are limited [163]. A modified pullulan was used for loading mitoxantrone. Pullulan nanoparticles hydrophobically modified with cholesterol were used to load mitoxantrone, and improved mitoxantrone delivery was observed using cholesterol substituted pullulan polymers (CHPs) [164]. The different kind of CHPs were synthesized on the basis of the degree of cholesterol substitution and diameter. The larger the size of the nanoparticles, the greater the drug release capacity will be. Drug-loaded CHP nanoparticles with the largest size showed more anti-cancer activity in bladder cancer cells, which was confirmed by flow cytometry. The drug-loaded nanoparticles could inhibit the migration of MB49 cells [165].

#### 4.8.3. Pullulan with Curcumin

**Figure 8:**
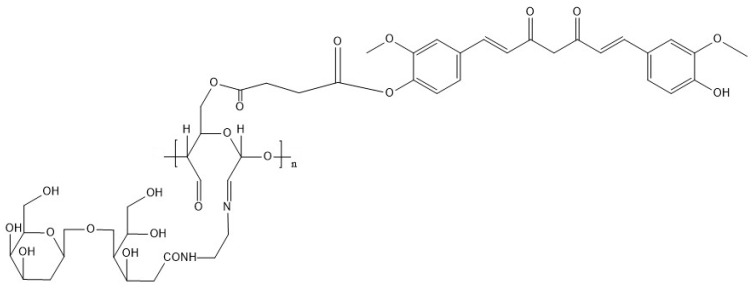
Structure of galactosylated pullan–curcumin conjugate.

Pullulan is hydrophilic in nature, so it could be used to deliver the hydrophilic substances, but not hydrophobic substances [166]. This problem could be resolved by forming pullulan acetate particles with amphiphilic nature by the process of acetylation. Pullulan acetate could be used as a carrier for delivering the drugs. The nanoparticle synthesis of curcumin-loaded pullulan acetate was performed to improve curcumin’s stability and physiochemical properties. It displayed improved biocompatibility and hemocompatibility in the embryo of zebrafish in vitro. The curcumin–pullulan acetate nanoparticle complex was used as a hepatoprotective agent. The conjugate enhances the solubility, pH stability, and photostability of curcumin [167]. A Galactosylated pullulan–curcumin conjugate (Figure 8) was synthesized for targeted delivery of curcumin to hepatocarcinoma. Galactosylated pullulan was conjugated with curcumin using succinic anhydride introducing acid functionalities. Galactosylated pullulan–curcumin conjugate shows higher toxicity and internalization towards HepG2 cells via asialoglycoprotein-mediated endocytosis compared to its non-galactosylated counterpart. The results suggest that the higher uptake of galactosylated pullulan–curcumin conjugate may take place through asialoglycoprotein receptor (ASGPR)-mediated endocytosis [168].

## 5. Potentials and Prospective of Polysaccharides in Cancer Drug Delivery

The utilization of traditional chemotherapy drugs was limited by the side effects and physiological barriers of drug delivery (blood circulation, tumor accumulation, tumor tissue penetration, endocytosis, and drug release) in the human body. The development of new materials and the modification of existing materials are the critical factors in constructing efficient carriers. Natural polysaccharides with good biocompatibility and unique physicochemical properties are considered ideal for drug delivery applications (Table 2). Meanwhile, choosing suitable polysaccharides for drug delivery poses several challenges. Polysaccharides with diverse functional groups enable chemical modification and help to conjugate or load desired drug compounds. The drug molecule can be released to the diseased site through simple diffusion or cleavable cross-linking. Polysaccharides possess biocompatibility and biodegradable properties, and they are often used as a suitable polymer matrix for designing new carriers for drug delivery. Polysaccharides can be quickly degraded under biological conditions and removed by renal clearance compared to other materials. In addition, polysaccharide-based carriers can easily be linked with targeting ligands aiming to deliver the drug to the diseased site without affecting the healthy cells. Drug leakage in the blood, resistance and prolonged blood circulation of the drug are the crucial factors to be considered while designing the carrier. Furthermore, the carrier should provide high encapsulation efficiency, and sustained drug release to achieve the synergistic effect.

## 6. Conclusions

Natural and synthetic polymer-based drug carriers take advantage of the unique delivery mechanism, such as target-specific delivery and prolonged circulation, by circumventing the immune systems. Application of biocompatible polymers, including their use as delivery systems for several potent anti-cancer drugs, are reported. This review highlighted the drug delivery applications of various polysaccharides, including alginate, dextran, chitosan, and hyaluronic acid. The polysaccharides with diverse functional groups can be modified with other chemical groups to make the conjugation of preferred therapeutic molecules easier. The drug moiety can be released through diffusion or by degradable cross-linking. Biocompatibility, biodegradability, and well-defined molecular weight are the crucial factors for a material to be used for therapeutic applications. Polysaccharides naturally possessing these properties can act as an appropriate polymer matrix for designing new materials. Polysaccharide-based nanomaterials have degraded into harmless derivatives under biological conditions and are easily removed from the body. Moreover, polysaccharides can be linked to targeting ligands such as folic acid to achieve effective drug delivery to the diseased site, provide cytotoxic specificity, and prevent toxic effects on healthy cells. Polysaccharide-based drug delivery systems can carry multiple drugs and deliver the drug by sequential or simultaneous release to achieve the synergistic effect. Polysaccharides loaded with proteins, peptides, drugs, and growth factors open up a new pathway for enhanced cancer treatment.

## Figures and Tables

**Table 1:** Polysaccharides used for drug delivery [36,37].

Polysaccharides	Sources	Physicochemical Properties	Applications and Benefits
Chitosan	Shells of crab, shrimp, and krill	Soluble in weak acids, mucoadhesive, reacts with negatively charged surfaces	Tissue regenerative medicine, pulmonary delivery, ionotropic gelation
Alginates	Marine brown algae	Water soluble, anionic coacervation with ions and polycations	pH-dependent swelling nontoxic, diffusion, erosion, in situ forming hydrogels
Cyclodextrin	Degraded starch derived from potato, corn, rice., etc.	Water soluble, nontoxic	Nanocarrier for controlled drug release, gene and drug delivery
Pullulan	Bacterial Homopolysaccharides produced from starch by *Aureobasidium pullalans*	Neutral polymer underivatized pullulan has high water solubility	Emulsifier sustained-release preparations
Hyaluronic acid	Vertebrate organisms	Biodegradable, bioactive, nonimmunogenic	Anti-cancer drug delivery, wound healing and skin regeneration
Dextran	Bacterial strains, cell-free supernatant	Neutral polymer, solubility depends on degree of polymerization	Colon-targeted delivery
Guar gum	Seeds of *Cyamopsis tetragonoloba*	Water soluble, non-ionic, galactomannan forms a thixotropic solution, stable at pH 4–10.5	Controlled release, colon-targeted release, thermoreversible
Pectin	Plant cell wall	Negatively charged molecule	In Situ gelling, sustained delivery, drug delivery in colorectal carcinoma

**Table 2:** Anti-cancer studies using polysaccharide–drug formulation.

Formulation	Model Used	Biological Changes	Reference
PTX-trimethyl chitosan conjugates	H22 tumor-bearing mice	Enhanced mucoadhesion and intestinal transport of PTX, increased tumor retardation and survival rate	[59]
Erlotinib-loaded MTX-chitosan magnetic nanoparticles	OVCAR-3 cell lines,	Improved cellular uptake, greater cytotoxicity and target specific delivery in FR-positive cancer cell lines	[64]
Curcumin-loaded chitosan nanoparticles	Swiss albino mice	Inhibiting the B[a]P-induced lung carcinogenesis, overexpression of p65 in the nuclei, reduced the overexpression of proliferating cell nuclear antigen	[67]
Curcumin-loaded folate-modified-chitosan-nanoparticles	MCF7 cell lines, L929 cell lines	Target specific uptake of curcumin into cancerous cells	[68]
Alginate nanoparticles with curcumin and resveratrol	DU145 prostate cancer cells	Increased cell uptake and enhanced cytotoxicity in cancer cells	[87]
EXE-loaded alginate nanoparticles	Dalton’s lymphoma ascites cells	Improved cytotoxicity	[90]
Ag/Alg-TMX-PEG/FA core shell nanocomposite	MCF7 cell lines	Inducting reactive oxygen species (ROS), downregulation of survival oncogenic genes, G2/M phase arrest	[93]
Pectin–curcumin composite	KYSE-30 cell lines	Release of curcumin from the composite at acidic pH, enhanced cytotoxicity	[101]
Dihydroartemisinin-loaded DOX–pectin conjugate	MCF-7 cell lines, C57BL/6 mouse	Intranuclear uptake in MCF-7 cell lines, significant reduction in tumor growth	[103]
MTX-loaded guar gum microspheres	Albino rats	Target specific delivery to the colon	[114]
Dextran–DOX micelles	Balb/C mice bearing 4T1 tumors	Acid-sensitive drug release minimize systemic toxicity in normal tissues Selective accumulation in tumor and enhanced tumor-suppressive efficiency	[122]
MTX-loaded dextran–CUR nanoparticles	MCF-7 cell lines	Rapid internalization and enhanced cytotoxicity	[133]
PTX–HA conjugate	MCF-7 cell lines BALB/c nude mice	Cellular internalization, and tumor targeting via CD44 caveolae-mediated endocytosis, target specific drug release in the presence of GSH	[137]
Paclitaxel-loaded cyclodextrin–polypeptide conjugates	MCF-7 and 4T1 cell lines	Enhanced cellular uptake, inhibit P-gp efflux pumps	[157]
Mitoxantrone loaded modified pullulan nanoparticles	MB49 cells	Inhibit the growth migration of MB49 cells	[164]

## Data Availability

Not applicable.
